# Fermented Food–Polysaccharides as Gut Health Regulators: Sources, Optimization, Structural Characteristics and Mechanism

**DOI:** 10.3390/foods14234108

**Published:** 2025-11-29

**Authors:** Aoxiang Zhou, Nanhai Zhang, Huanhuan Dong, Yousheng Huang, Liuming Xie

**Affiliations:** School of Pharmacy, Jiangxi University of Chinese Medicine, Nanchang 330004, China; zhouaoxiang@jxutcm.edu.cn (A.Z.); zhangnanhai@jxutcm.edu.cn (N.Z.); donghh@jxutcm.edu.cn (H.D.); huangyousheng@jxutcm.edu.cn (Y.H.)

**Keywords:** fermented, polysaccharides, optimization, structure, gut microbiota, intervention mechanisms

## Abstract

Polysaccharides are natural macromolecules with significant functional properties, but the application of certain natural polysaccharides is hindered by their large molecular weight, poor solubility, and limited functionality. In recent years, microbial fermentation has emerged as a sustainable, low-energy-consuming and low-pollution green biotechnology strategy, which degrades and modifies polysaccharides by generating carbohydrate-active enzymes, thereby obtaining new types of polysaccharides with lower molecular weights and stronger functions. Meanwhile, fermented polysaccharides are utilized as prebiotics by intestinal microorganisms. By regulating microbial communities and their metabolites (such as short-chain fatty acids and bile acids), fermented polysaccharides have shown potential value in maintaining metabolic homeostasis and intervening in related diseases. Based on the results of the latest research, this paper summarizes the sources, optimization of fermentation conditions, structural characteristics of fermented food–polysaccharides, with the aim of providing new insights into the utilization of polysaccharides. Meanwhile, focuses on discussing the effects of fermented polysaccharides on the gut microbiota and the mechanisms by which they intervene in disease through regulating the microbiota and its metabolites, which offered new insights and directions for the therapeutic application of fermented food—polysaccharides.

## 1. Introduction

The gastrointestinal tract, the greatest immune organ, bears a profound relationship with human well-being, which can be divided into four layers of barriers: biological, chemical, mechanical, and immune [1]. The gut microbiota belongs to the biological barrier, which plays a crucial role in maintaining health [2]. This microbial community contains over 1000 species of microorganisms, with bacteria forming the majority, including the phyla *Firmicutes*, *Bacteroidetes*, *Proteobacteria*, and *Actinobacteria* [3,4]. Studies show that beneficial bacteria (such as *Bifidobacterium*, *Lactobacillus*) can aid digestion, synthesize vitamins, and metabolize to produce short-chain fatty acids (SCFAs), while harmful bacteria (such as *Enterococci* and *Clostridium*) can cause adverse reactions such as inflammation when they proliferate abnormally. In a healthy host, beneficial and harmful bacteria maintain a dynamic balance to keep the gut stable [1,5]. Gut microbiota provide energy to intestinal epithelial cells by metabolizing polysaccharides and polyphenols. Concurrently, polysaccharide-derived pH regulation limits the colonization of pathogenic bacteria, thereby triggering immuno-regulatory effects [6]. Meanwhile, beneficial bacteria actively utilize microbiota-derived metabolites (SCFAs, bile acids, and other), thereby promoting an increase in their abundance. More importantly, these metabolites can act on organs such as the intestines and liver, thereby activating the corresponding pathways and slowing down the corresponding disease symptoms [7,8]. For example, butyric acid restores immune homeostasis by inhibiting Histone deacetylase (HDAC activity), blocking the pro-inflammatory interleukin-6 (IL-6)–signal transducer and activator of transcription 3 (STAT3)–interleukin-17 (IL-17) inflammatory axis, and promoting T cells (Tregs) differentiation. Simultaneously, it upregulates tight junction proteins and mucins to repair the intestinal barrier and alleviate gut inflammation [9,10]. Therefore, preserving microbiome homeostasis and stimulating metabolite biosynthesis is a keynote regulator in improving a variety of diseases, including metabolic diseases, intestinal diseases, and immune diseases [5].

Some natural polysaccharides are biologically active macromolecules with multiple functions (such as anti-aging, antioxidant, anti-inflammatory, and immunomodulatory effects), offering the advantages of being mild and safe [3,4,7,11]. Numerous studies have shown that polysaccharides can have a positive influence on human wellness through the regulation of the gut flora and its metabolites [12,13,14,15]. *Schisandra sphenanthera* polysaccharides (SSP) could significantly reduce the competitiveness of harmful bacteria such as *Proteobacteria* and *Escherichia-Shigella*, while concomitantly promoting the proliferation of beneficial bacteria such as *Enterococcus* and *Bacteroides* [16]. Sea buckthorn polysaccharides reversed the intestinal flora imbalance in mice caused by cefixime by upping the proportion of *Akkermansia*, *Faecalibaculum* and *Allobaculum* and suppressing the colonization of *Helicobacter* [17]. However, some natural polysaccharides have limitations in their application as prebiotics ascribable to their high molecular mass, limited aqueous solubility, and suboptimal functionality. Therefore, it is particularly important to develop new extraction methods that enhance the yield and activity of polysaccharides.

In recent years, with the continuous improvement of requirements for polysaccharide yield and biological activity, researchers have gradually recognized that microbial fermentation is an effective way to increase the yield and activity of polysaccharides. The number of related studies has continued to grow, and polysaccharide extraction strategies based on microbial fermentation have gradually developed [18,19]. Studies have shown that fermented food–polysaccharides often exhibit stronger biological activity, which offers enormous promise for application in functional food development and the pharmaceutical industry [20,21,22,23,24,25]. For example, polysaccharides fermented from asparagus by *Lactobacillus plantarum* NCU116 exhibit stronger immune activity and antioxidant activity [20]. Compared with unfermented *Polygonatum kingianum* polysaccharides (23.67 ± 0.76 d), the fermented polysaccharide (26.06 ± 1.73 d) showed a significant difference in extending the lifespan of *C. elegans*, with a relative increase of 10.09%, and also demonstrated enhanced efficacy in improving aging phenotypes such as motor ability and lipofuscin accumulation [21]. Fermented *Astragalus* polysaccharides exhibited superior antioxidant capacity [22]. For example, polysaccharides produced by the fermentation of *Hericium erinaceus*, *coix* seed, and *Sporidiobolus pararoseus* [23,24,25]. Therefore, as researchers delve deeper into the field of fermented foods, there is amounting recognition of the importance of exploring changes in polysaccharide structure and biological activity during microbial fermentation. Although previous studies have confirmed that fermentation can significantly alter the molecular weight, glycosidic bonds, and branch chain configurations of polysaccharides, thereby influencing their biological activity, systematic summaries of the structural modification patterns induced by different strains and enzyme systems remain lacking. Furthermore, research on how to selectively enhance polysaccharide activity through process parameter optimization remains fragmented. More critically, existing research predominantly focuses on how polysaccharides promote health by modulating gut microbiota and their metabolites. However, the specific mechanisms by which they alleviate specific diseases through regulating the interactions between gut microbiota and metabolites have yet to be systematically and deeply explored.

Consequently, this paper systematically reviews the sources of polysaccharides in fermented foods, summarizes how different microbial strains and fermentation conditions regulate polysaccharide structure and physicochemical properties, and introduces strategies for condition optimization. It establishes a systematic framework spanning strain selection, process optimization, structural remodeling, and functional expression, providing a theoretical basis for the large-scale production and targeted modification of polysaccharides. Furthermore, this paper explores the interactions between fermented food–polysaccharides and the gut ecosystem, summarizing potential pathways through which fermented polysaccharides participate in disease regulation by modulating the gut microbiota and its metabolites. This review provides a basis to further investigate the fermented polysaccharides, which is beneficial to further develop the application of polysaccharides.

## 2. Source

**Figure 1:**
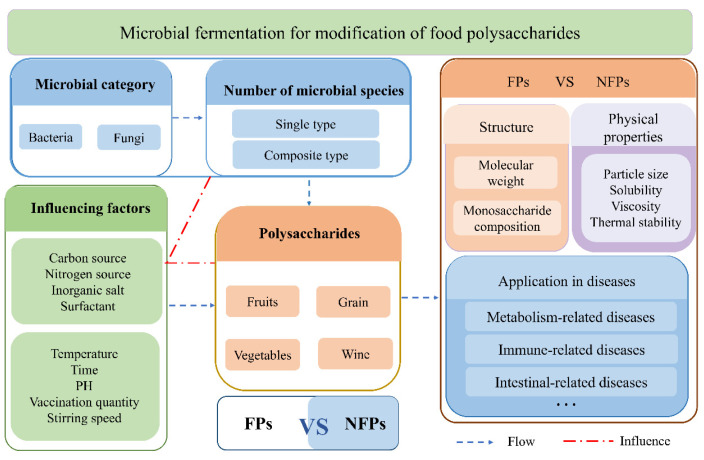
Research guidelines for polysaccharides in microbial fermented foods: Firstly, classify the fermentation system according to the type of microorganisms and the quantity of species; Then, the influence of substrate category and key process/chemical factors on the modification effect is discussed; Finally, a systematic comparison of polysaccharides was conducted before and after fermentation, quantitatively evaluating their structural characteristics, physicochemical properties and application in diseases. Based on this, the differences between fermented polysaccharides (FPs) and unfermented polysaccharides (NFPs) were clarified.

The acquisition and modification of polysaccharides in fermented foods are influenced by multiple factors (Figure 1). This process not only reshapes the structural characteristics of the polysaccharides, but also directly determines the yield and quality. Among them, the selection of specific microorganisms is the primary threshold: the carbohydrate enzyme (CAZymes) systems and metabolic characteristics carried by different bacterial groups are different, which determine the degree and direction of modification of the polysaccharide structure, thereby influencing their biological activity [8]. Based on this, the representative microorganisms involved in the fermentation of food polysaccharides are presented below from the perspectives of bacteria and fungi, respectively.

### 2.1. Bacterial Fermentation Food–Polysaccharides

At the bacterial level, lactic acid bacteria (LAB) utilize an exo-acting/deglycosylating repertoire of CAZymes to achieve, under mild conditions, moderate polysaccharide depolymerization, debranching, and enhanced solubilization. These actions convert insoluble or poorly soluble fractions into soluble, oligosaccharide-rich fractions, enabling gentle and selective modification in end-product matrices such as doughs and fruit-/vegetable-based systems [26,27]. For example, the utilization of α-amylase-mediated hydrolysis by *Lactobacillus plantarum* CICC 21790 in a cereal matrix renders starch granules more brittle, lowers the gelatinization temperature, and reduces the final viscosity [28]. *Lactobacillus plantarum* RI 111 secretes a cellulolytic/hemicellulolytic enzyme system to degrade polysaccharides in agro-industrial by-products (such as straw, molasses, palm kernel cake, and soymilk), which includes endoglucanase, exoglucanase, β-glucosidase, and mannanase [29]. *Lactiplantibacillus plantarum* ATCC 14917 primarily cleaves the β-(1→4) backbone of litchi water-insoluble polysaccharides (WISP) via endo-β-1,4-glucanase, while side-chain glycosidases (β-glucosidase) cooperatively debranch and solubilize the material, thereby converting WISP to water-soluble polysaccharides (WSP); the weight-average molecular weight (*Mw*) of the resulting WSP decreases from 578.23 to 421.47 kDa [30]. *Lactiplantibacillus plantarum* 84-3 employs β-glucosidase and α-amylase to selectively degrade resistant starch and plant polysaccharides, collectively improving solubility and flavor [31]. In addition, *Lacticaseibacillus paracasei* B41 presents cell-surface β-fructofuranosidase that selectively hydrolyzes the β-(2→1) linkages of inulin to release fructose; in synergy with amylolytic enzymes, this establishes a parallel utilization pathway for inulin and starch, accelerating fructo-oligosaccharide breakdown and lactic acid production, thereby enhancing the flavor profile of fermented vegetable products [32].

*Bacillus* spp. can secrete endolytic hydrolases (such as α -amylase, xylanase, β-1,3/1, 4-glucanase, pectinase), which pre-treat and rapidly saccharify the polysaccharides in raw materials/by-products, causing structural chain cleavage and thereby enhancing solubility. Legumes, potatoes, corn, and their byproducts are frequently used as polysaccharide fermentation substrates [33]. For example, *Bacillus licheniformis* is often used in the fermentation of polysaccharides in foods such as legumes, which mainly degrades cell wall-related polysaccharides by secreting polysaccharide hydrolases like α-amylase and β-glucosidase, thereby enhancing the solubility and fermentability of the substrate [34]. In cassava-based fermentations, *Bacillus* species use the same enzymatic toolkit to lower matrix viscosity, release fermentable sugars, and concomitantly improve product texture and flavor [35].

### 2.2. Fungal Fermentation Food–Polysaccharides

Fungal fermentation leverages filamentous fungi (such as *Aspergillus*, *Rhizopus*, *Mucor*) as well as yeasts/non-Saccharomyces yeasts. These microbes are characterized by the secretion of broad-spectrum extracellular CAZymes consortia (including cellulases, hemicellulases, pectinases, and oxidative enzymes), which confer wide substrate coverage. Therefore, they excel at deep depolymerization and solubilization of complex plant cell-wall polysaccharides, making them well suited for substantial structural remodeling and solubility enhancement [36]. In fruit wine and grape pulp systems, non-yeast fungi and yeast fungi (such as *Aureobasidium pullulans* and *Nakazawaea ishiwadae*) abundantly produce pectinase clusters and β-glucosidase, targeting primarily pectin and glycoside-type aroma precursors to enhance juice yield, improve clarity, and release aromatic ligands [37]. In amylolytic starter cultures, *Aspergillus*, *Rhizopus* and *Mucor* species commonly employ α-amylase (GH13) glucosidase (GH15), and debranching enzymes as core components, supplemented by hemicellulases. These enzymes saccharify and undergo primary degradation of amylose, amylopectin, and cell wall polysaccharides, providing fermentable sugars for subsequent fermentation [38]. During the early stage of tea pile-fermentation, filamentous fungi (particularly *Aspergillus*) predominate. Their broad-spectrum extracellular CAZymes consortia (cellulases, hemicellulases, pectinases, and oxidative enzymes), together with starch-degrading α-amylase (glycoside hydrolase family 13, GH13) and glucoamylase (GH15), deeply depolymerize and solubilize cell-wall polysaccharides and starch granules, lowering the total polysaccharide content from 28.30 to 11.86 mg/g and releasing soluble sugars such as glucose (Glc), fructose (Fru), and glucosamine (GlcN). As fermentation proceeds to the late stage, the community shifts toward lactic acid bacteria and *Bacillus*, whose acidification and endolytic enzyme systems drives polysaccharide content up to 54.90 mg/g, accompanied by an increase in structural monosaccharides (including galactose (Gal), rhamnose (Rha), and mannose (Man)), which is consistent with polysaccharide remodeling and accumulation [39]. Overall, the fungal fermentations are particularly well suited to deep structural remodeling and solubilization within food–polysaccharide matrices for its broad-spectrum extracellular enzyme activity.

In summary, bacteria focus more on the mild directional modification of polysaccharides/pretreatment saccharification, while fungi achieve deep depolymerization and solubilization through broad-spectrum extracellular CAZymes. In practice, a series path of fungal pretreatment-bacterial refinement can be adopted to balance efficiency and structural controllability. In recent years, it has been proposed to use food-grade /GRAS hosts as the “chassis” to promote the controllable transformation of fermented foods by tailoring CAZymes spectrum and metabolic flux according to predefined polysaccharide structural functional endpoints (such as *Mw*/DP reduction, side chain removal/debranching, and solubility improvement). Based on this, microorganisms with key CAZymes activities are selected or engineered to achieve targeted modification and controllable yield [40,41]. However, a critical gap remains: the absence of systematic comparative studies conducted under standardized conditions (e.g., using identical substrates, processing protocols, and unified evaluation metrics for chemical composition, enzymatic fingerprinting, rheological/volatility characteristics and functional activity). This deficiency makes it difficult to quantitatively determine the unique contributions of different microbial strains or enzyme profiles to structure-function transformations.

## 3. Optimization of Fermentation Conditions

After the microorganisms are selected, the composition of the culture medium and the fermentation conditions affect the yield, structure and bioactivities of polysaccharides by influencing the fermentation microorganisms. This section discusses the influence of medium components and fermentation conditions on the yield of polysaccharides. Meanwhile, the optimal fermentation conditions for several polysaccharides are summarized (Table 1).

### 3.1. Optimization of Culture Medium Components

The culture medium is the basis for microbial fermentation to produce polysaccharides, which is closely related to the yield and quality of fermented food–polysaccharides [47,48]. Carbon sources are an essential component of culture media, providing energy for microbial growth and food–polysaccharide modification. Fermented food–polysaccharides are typically derived from plants rich in target polysaccharides, which serve as either the primary carbon source or an auxiliary carbon source. Different contents of carbon sources greatly impact on microbial metabolism and polysaccharide yield. Yang et al. investigated the effect of different addition levels (1%, 3%, 5%, 7%, 9%, 11%, and 20%) of *Astragalus* as the sole carbon source on polysaccharide yield [44]. The results indicated that the polysaccharide yield was highest when the addition level was 9%. In addition, the use of corn stover and cassava sawdust not only provides the carbon source required for mycelial growth but also continuously supplies sugars derived from the decomposition of lignocellulose through the activated hydrolytic enzyme system. This process enhances polysaccharide synthesis, increasing polysaccharide yields in these substrates [49]. Nitrogen sources are one of the key factors influencing polysaccharide yield, which are commonly categorized into organic nitrogen sources (such as peptone and yeast extract) and inorganic nitrogen sources (such as ammonium sulfate and potassium nitrate). Different strains exhibit specific preferences for nitrogen source type and concentration. These variations alter carbon flux allocation, thereby influencing polysaccharide synthesis levels and yield [50]. Additionally, trace elements in the medium (such as iron and zinc), acting as cofactors for metabolic enzymes, can indirectly regulate polysaccharide biosynthesis [51]. Surfactants strengthen microbial cell membrane permeability, thereby stimulating the secretion of fermentative polysaccharides [52]. Span 80 increased membrane permeability in a dose-dependent manner, thereby enhancing the secretion flux of *Tremella fuciformis* polysaccharide. Under this condition, the yield was maximal (22.51%) and the glucuronic acid (GlcA) content was increased, concomitantly strengthening radical-scavenging activity against hydroxyl OH and DPPH radicals [52]. Yang et al. used *L. plantarum* M616 fermentation. When adding peptone (1.0 g/L), yeast extract (1.0 g/L), MgSO_4_ (1.0 g/L), and KH_2_PO_4_ (1.0 g/L) in a 10% (*w*/*v*) yam solution, the polysaccharide yield was increased from 71.03% ± 2.75% to 76.28% ± 2.37% [53].

### 3.2. Optimization of Fermentation Conditions

Fermentation conditions include temperature, time, pH, stirring speed, inoculum amount, etc. These conditions are interrelated and collectively influence the yield and quality of fermented food–polysaccharides. Temperature is critical during fermentation, as it not only affects microbial growth but also enzyme activity, which in turn affects the yield of fermented polysaccharides. For example, high temperatures can cause moisture imbalance and rapid metabolism, causing significant damage to microorganisms. This reduces the yield of fermented polysaccharides and may alter their structural properties [54,55]. Fermentation time is closely related to the growth and metabolism of microorganisms. In the early stages of fermentation, microorganisms need to adapt to the environment and produce small amounts of polysaccharides. When microorganisms enter the logarithmic growth phase, they secrete large amounts of enzymes to break down plant cell walls, thereby promoting an increase in the yield of polysaccharides [47]. PH not only affects the permeability of microbial cell membranes, but also affects enzyme secretion and the activity of extracellular enzymes. Furthermore, pH may also affect the form of fermentation substrates, thereby altering the structure of polysaccharides [56]. Stirring speed also affects polysaccharide yield. Stirring speed affects dissolved oxygen levels and the uniformity of the fermentation substrate [46]. Inoculum density critically determines fermented polysaccharide yield. For example, as the inoculum size increases from low to high, the yield of polysaccharides first increases and then decreases. This may be because an increase in inoculum size shortens the lag phase, thereby increasing enzyme yield. However, if the inoculum is too large, available metabolic substances are primarily used to sustain self-metabolism, thereby limiting the synthesis of extracellular decomposing enzymes and ultimately leading to a decrease in fermented polysaccharide yield [18]. When these conditions interact to achieve optimal conditions, the yield of fermented food–polysaccharides is high.

The outcome of fermentation is not determined by a single factor, but rather the result of the synergy or antagonism of multiple factors. This is particularly evident in the “carbon × nitrogen” interaction: altering the type or amount of nitrogen source often leads to changes in the optimal carbon source selection, with the optimal solution appearing as “paired combinations” rather than a single fixed carbon source. For example, during the optimization of the fermentation medium for *Sanghuangporus alpinus* (all other conditions being equal), the optimal combination of glucose and yeast extract was 4.1–24.6 g/L and 7.8–7.9 g/L, respectively. Notably, When the yeast extract is decreased, the optimal point of glucose shifts to the “right” side of the high concentration, suggesting that a higher carbon source is needed to compensate for the decline in enzyme expression and secretion flux caused by nitrogen restriction [57]. In terms of process parameters, there is a significant combined effect between time and the feeding rate. For example, response surface combination optimization of *Aspergillus niger* fermentation of *Artemisia* polysaccharides revealed a significant interaction between time and feed rate [46]. Under higher feed conditions, matching an appropriate fermentation duration is essential to achieve higher extraction rates with insufficient time leading to inadequate release, while excessive time may cause a decline due to product inhibition or enzyme inactivation. Similarly, temperature × inoculum concentration exhibited an antagonistic relationship in the response surface optimization of *Trichoderma viride* fermentation for *Auricularia auricula* polysaccharide production. At an inoculation level of 10%, increasing the temperature from 29 to 31 °C resulted in a 3.6% increase in degradation rate, whereas at an inoculation level of 20%, the same temperature change (29 to 31 °C) led to an 8.9% decrease in degradation rate, indicating that these parameters cannot be optimized by simply changing them in the same direction but must instead be tuned in combination [43]. Furthermore, temperature × pH exhibited a synergistic effect: under identical conditions, simultaneously increasing temperature and pH enhanced extraction efficiency. At 30 °C and pH 5, the yield of *Angelica sinensis* polysaccharide reached 15.35% [58]. Mechanistically, these interactions share consistent enzyme-substrate rationales: when pH is stabilized near the optimal range, more hydrolases exist in the correct dissociated state with greater thermal stability, allowing moderate temperature increases within this range to enhance catalytic rates. Concurrently, an optimal pH promotes dissociation and site exposure of acidic groups in plant cell walls and polysaccharides, while elevated temperature enhances chain segment mobility and diffusion. The combined effect increases accessibility × catalytic efficiency, forming a positive synergy between temperature and pH. Conversely, early metabolic heat release and mycelium network thickening increase apparent viscosity at high inoculum levels. At this stage, further temperature elevation may accelerate enzyme rates but trigger product inhibition, resulting in an antagonistic relationship between temperature and inoculum level [43]. Therefore, the optimization of conditions for polysaccharide fermentation should be carried out under the framework of multi-factor coupling.

### 3.3. Methodology of Condition Optimization and Fermentation Amplification Strategy

In optimizing fermentation conditions for polysaccharides, a multivariate process involving screening, modeling, and validation is strongly recommended. While single-factor methods may initially explore parameter ranges, they are time-consuming and incapable of revealing factor interactions or true optimal points, making them unsuitable as final optimization tools. Subsequently, a two-level screening design (such as Plackey–Berman) identifies key factors from multiple variables. This is followed by the response surface methodology (RSM) stage, where quadratic polynomials explicitly characterize main effects, curvature, and interaction terms. A 3D surface plot is then used to search for paired optimal conditions under combined effects [59]. When strong nonlinearity or “super-quadratic” behavior is present, artificial neural networks combined with genetic algorithms (ANN–GA) can serve as a complementary approach. In the control study of xylan fermentation, the optimal conditions obtained by ANN-GA were similar to those derived from RSM. ANN–GA yielded optimal conditions similar to those from RSM. However, ANN–GA’s optimal yield prediction error was approximately 2% compared to about 8% for RSM. This indicates its superiority in nonlinear global optimization and prediction, while RSM offers a more straightforward approach for directly identifying interactions [60]. It is important to note that the transition from laboratory-scale shaking flasks to industrial fermenters is not a direct conversion. During scale-up, the fermentation system’s oxygen supply, mixing time, shear force, and heat transfer capacity all change. Significant interactions observed in the laboratory, such as “temperature × pH,” “temperature × inoculum level,” and “time × feed rate,” often alter under tank-scale conditions. Therefore, conditions determined via RSM/ANN must be validated and calibrated under constraints identical to those of industrial fermentation [61]. Simultaneously, the value and cost of polysaccharides derived from food sources dictate the goal of synergistically reducing costs through strain selection, raw materials, and process optimization. Therefore, during scale-up, factors such as raw material costs, energy consumption, anti-foaming agents, alcohol precipitation, and recovery rates should be integrated into the optimization process to achieve sustainability and profitability as multiple objectives [62]. In summary, the transition of fermented food polysaccharides from laboratory fermentation flasks to industrial fermentation tanks should be advanced through coordinated efforts in optimizing methods, achieving engineering equivalence, ensuring economic sustainability. This approach will ultimately establish replicable and scalable fermentation strategies.

## 4. The Effect of Fermentation on Structure and Physical Properties of Food–Polysaccharides

After determining the selection of microorganisms and optimizing conditions, the fermentation process alters the *Mw*, monosaccharide composition, and physicochemical properties of food–polysaccharides (Table 2).

### 4.1. Effect of Fermentation on Molecular Weight of Polysaccharides

Microbial fermentation processes have a significant impact on polysaccharide structures, including changes in molecular weight [8]. Hu et al. conducted structural analysis on the purified unfermented longan polysaccharides (P1) and fermented longan polysaccharides (P2, P3, P4), revealing that the *Mw* of P2, P3, and P4 (76.47 ± 3.25 kDa, 36.77 ± 0.52 kDa, and 41.83 ± 2.05 kDa) were notably lower than that of P1 (3345.26 ± 22.31 kDa) [19]. The *Mw* of *Polygonatum kingianum* polysaccharides was decreased significantly before and after fermentation [21]. *Lactobacillus gasseri* JM1 fermentation reduced the *Mw* of *Hericium erinaceus* polysaccharides from 7.5 × 10^7^ Da to 3.5 × 10^4^ Da [63]. This significant decrease is speculated to be related to the strain’s CAZymes, which enable it to utilize polysaccharides. The same results were obtained from research on fermented carrots. This change may be caused by microbial production of endo-/exo-glucanases and mannanases hydrolyzing β-1,4- and β-1,6-linkages [64]. During the fermentation of lychee pulp by *Lactiplantibacillus plantarum* ATCC 14917, research revealed that microorganisms produce glycoside hydrolases such as endoglucanase and β-glucosidase, which directly catalyze the cleavage of insoluble polysaccharides (WISP), leading to the accumulation of water-soluble polysaccharides (WSP). This process is accompanied by a decrease in WSP *Mw* from approximately 578 kDa to 421 kDa [30]. However, the *Mw* of *Lentinus edodes* polysaccharides actually increases during lactic acid bacteria fermentation. The observed rise in *Mw* after fermentation does not necessarily indicate polymerization. It may also result from fermentation breaking down cell walls and dissociating the matrix (proteins, starch, fiber), allowing previously insoluble high-*Mw* fragments to enter the solution phase and be detected. Additionally, fine-tuning of branching or supramolecular conformation may increase hydrodynamic volume, leading to a larger apparent *Mw* in size exclusion chromatography/size exclusion gel filtration chromatography (SEC-RI). Furthermore, extracellular polysaccharides (EPS) may be incorporated. Therefore, the “increase/decrease” in *Mw* is not a unidirectional pattern but is determined by the combined effects of substrate structure, strain enzyme profile, and process conditions [65].

### 4.2. Monosaccharide Composition

The fermentation process utilizes CAZymes such as exoglycosidase to catalyze trans-substitution reactions, thereby achieving selective rearrangement of the monosaccharide composition and molar ratio of polysaccharides. A comparison of monosaccharide components before and after fermentation of *Polygonatum kingianum* revealed that the main monosaccharides in unfermented *Polygonatum kingianum* polysaccharides (NFP) were galactose and arabinose, while the main monosaccharides in fermented *Polygonatum kingianum* polysaccharides (SFP) were mannose, galacturonic acid, galactose, and arabinose [42]. Additionally, testing of fructose content revealed that fermentation significantly reduced fructose levels, with SFP showing a 19.2% reduction in fructose content compared to NFP. Both *Lycium barbarum* polysaccharides (LBP) and fermentation *Lycium barbarum* polysaccharides (FLBP) are composed of eight monosaccharides: mannose, glucuronic acid, rhamnose, galacturonic acid, glucose, galactose, arabinose, and fructose [66]. However, there is a marked difference in the molar ratio of monosaccharide composition between LBP and FLBP. Specifically, the molar ratio of monosaccharides in LBP is 1.28:0.46:0.64:1.16:1.50:4.62:5.73:0.62, while the molar ratio of monosaccharides in FLBP is 2.98:1.35:1.27:3.12:1.54:9.01:10.53:3.47, which indicates that fermentation treatment significantly altered the ratio of monosaccharide components in the polysaccharide, resulting in a noticeable change in the molar ratio of each monosaccharide component in FLBP compared to LBP. Changes in monosaccharide composition can result from the specific hydrolysis of terminal groups at branching sites by exo-glycosidases (such as α-L-arabinofuranosidase, β-galactosidase, β-glucosidase). Concurrently, the preferential metabolism of specific monosaccharides/oligosaccharides by microorganisms further alters the proportion of monosaccharides. Cheng et al. compared the structure of fermented *Tetrastigma hemsleyanum* Diels et Gilg polysaccharides (F-THDP2) with that of unfermented *Tetrastigma hemsleyanum* Diels et Gilg polysaccharides (THDP2), revealing that fermentation reduced the *Mw* and significantly altered the monosaccharide composition. In THDP2, glucose was the main monosaccharide, while in F-THDP2, glucose was metabolized and converted into galactose, with no glucose present. Additionally, fermentation also altered the polysaccharide backbone. The backbone of THDP2 was →4)-α-d-Glcp-(1→ and →4)-β-d-Gal p-(1→, while the backbone of F-THDP2 is → 2)-α-d-Gal p-(1→ and →2)-α-d-Man p-(1→ [67]. Compared to jackfruit pulp (JP) polysaccharides, the glucuronic acid content in jackfruit pulp (JP-F) polysaccharides fermented with *Lactobacillus plantarum* FM17 was significantly reduced. When endonucleases such as polygalacturonase (PG) and pectin lyase (PL) acted on HG/RG-I, they cause depolymerization of the GalA units. These released units are then further metabolized, resulting in a decline in the measured GalA ratio [68]. The glucuronic acid content of fermented blue honeysuckle polysaccharides (BHP-L) was markedly higher than that of unfermented blue honeysuckle polysaccharides (BHP-U) [69].

### 4.3. Physical and Chemical Properties

Fermentation can transform polysaccharides from large, smooth-surfaced thin sheets into smaller, edge-broken fragments, suggesting granulation and an increase in specific surface area [63]. A decrease in *Mw* is usually accompanied by a reduction in viscosity. Meanwhile, the reduction in particle size and the increase in hydrophilicity/charge density (such as the rise in the proportion of aldehyde acid) jointly drive the enhancement of solubility and dispersion stability [70]. For example, *Lactobacillus delbrueckii bulgaricus*-fermented *Dendrobium officinale* polysaccharide (F-DOP) exhibited higher aqueous solubility, plausibly attributable to a fermentation-induced transition to a finer, more compliant (fragmented/porous) microstructure [71]. At a concentration of 3% (*w*/*v*), the viscosity of fermented Tartary buckwheat polysaccharide (FBP) was lower than that of the non-fermented counterpart (NBP) across the entire shear range, while its solubility increased from 74.17 to 80.91 mg/mL [72]. Thermal stability of NBP also was improved, with the decomposition temperature rising from 292.09 °C to 311.12 °C. These effects are plausibly attributable to the removal of low-molecular-weight impurities and thermolabile substituents (such as acetyl and methyl-ester groups), together with secondary rearrangement of interchain hydrogen bonding, which collectively delay thermal scission. Fermentation often leads to a decrease in the apparent viscosity of polysaccharide solutions. For instance, fermented *asparagus* polysaccharides (FAOP) showed a reduction in particle size and viscosity [73]. Similar trends are observed for fermented lily polysaccharides [74]. Mechanistically, these effects are attributable to decreases in *Mw* and particle size, which lower chain entanglement density, with potential contributions from changes in substitution and surface charge. Fermentation also affects the zeta potential of polysaccharides. For example, while the zeta potential magnitude of Low-temperature freeze–thaw *Eucheuma spinosum* polysaccharides (L-ESP-3) was increased with rising concentration, enhancing solution stability. Fermented Eucheuma spinosum polysaccharides (F-ESP) exhibited the opposite trend, with its zeta potential decreasing as concentration increased, leading to a slight reduction in stability. These differential changes are primarily influenced by the opposing effects of two mechanisms: surface charge generation (increased exposure of uronic acid groups) and charge shielding/adsorption (increased ionic strength, counterion coagulation, protein /EPS adsorption) [75]. In conclusion, fermentation process typically reduces polysaccharide *Mw* and particle size of polysaccharides, increases hydrophilicity/surface charge, and transforms dense, large-sheet structures into finer, porous fragments, thereby decreasing apparent viscosity while simultaneously enhancing solubility, dispersibility, and thermal stability. Meanwhile, shifts in zeta potential are matrix- and condition-dependent, reflecting the interplay between surface-charge generation and charge screening/adsorption.

**Figure 2:**
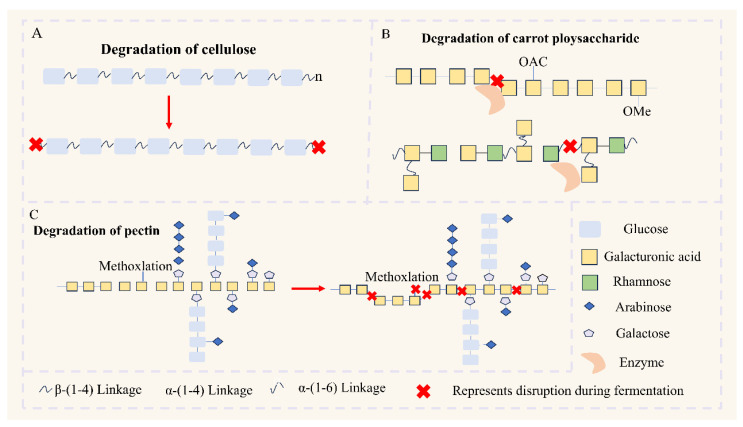
The degradation effect of fermentation on the food–polysaccharide structure. (**A**) The degradation effect of fermentation on cellulose structure. (Microbial cellulases hydrolyse β-(1→4) glucosidic linkages in the linear glucan backbone, leading to shorter chains.) (**B**) The degradation effect of fermentation on carrot structure. (Simplified model of the pectin-rich cell-wall polysaccharides in carrot. Pectinolytic enzymes cleave selected glycosidic bonds and remove O-acetyl (OAc) and O-methyl (OMe) ester substituents (red crosses), producing shorter and less substituted fragments.) (**C**) The degradation effect of fermentation on pectin structure. (Conceptual representation of homogalacturonan/rhamnogalacturonan regions indicates that fermentation can alter the degree of methoxylation and depolymerise the galacturonic-acid backbone and neutral-sugar side chains.

Collectively, fermentation modifies the *Mw*, monosaccharide composition, and physicochemical properties of polysaccharides. Additionally, fermentation can modify the fine structure of polysaccharides through CAZymes (Figure 2). For example, microorganism like yeast can hydrolyze starch or starch- derived oligosaccharides to yield glucose [76]. *L. plantarum* NCU 116 could partially degrade carrot polysaccharides by cleaving the 1,4- GalpA linkage between repeating units (→ 4)-α-α-GalpA-(1 → and → 2)- α-Rhap-(1 → 4)-α-GalpA-(1 →) in homogalacturonans and rhamnogalacturonan-I [64]. Yeast fermentation partially degrades pectin structures and modifies pectin, resulting in fermented apple pomace with lower homogalacturonans (HG) and degree of methoxylation as well as higher rhamnogalacturonan-I (RG I) [77]. These alterations reshape the role of polysaccharides in the digestion, absorption, and metabolism of the gastrointestinal tract, thereby exerting a series of effects on mucosal function and host health. In parallel, structural features govern the selective utilization of polysaccharides by the gut microbiota, thereby modulating community composition and key metabolites (SCFAs and bile acids) with system-wide physiological effects. Although many studies infer the enzymes involved by analyzing CAZymes genes, clear associations between specific enzymes and the measured functions remain limited. To close this gap, future work should combine targeted enzyme activity/inhibition assays with secretome/transcriptome profiling guided by CAZymes annotation and substrate localization studies, thereby connecting macroscopic observations (such as *Mw* reduction and compositional reprogramming) to the precise glycosidic linkages that are cleaved.

**Table 2:** Effects of fermentation on the physical properties, structure, and biological activity of polysaccharides.

Microbial Type	Microorganisms	Polysaccharides	*Mw*	Monosaccharide Composition	Physical Properties	Enhanced Bioactivities	Refences
Bacterial	*Lactobacillus plantarum* NCU116	*Momordica charantia* L. polysaccharides	↓	Galactose ↑ (29.71–34.99%), Glucose ↓ (14.24–10.48%), Mannose ↓ (14.24–1.85%)	Viscosity ↓	Antioxidant activity, anti-diabetic activity	[78]
	*Lactobacillus plantarum* NCU137	*coix* seed polysaccharides	↓	Glucose ↑ (68.61–72.91%)		Immunomodulatory activity	[24]
	*Lactobacillus plantarum* M616	Chinese yam polysaccharides	↓	Glccose ↑ 3.34%, Rhamnose ↓ 34.3%, Arabinose ↓ 48.4%, Galactose ↓ 15.0%, Mannose ↓ 15.6%		Anti-inflammatory activity	[53]
	*Bacillus* sp. DU-106	*Dendrobium officinale* polysaccharide	↓	Mannose ↑ (35.65–53.97%)		Immunomodulatory activity	[79]
	*Lactobacillus fermentum* 21828	*Lentinus edodes* polysaccharides	↑	Glucose ↑ (45.94–48.16%)		Immunomodulatory activity	[65]
	*Lactobacillus plantarum*	Lanzhou Lily Bulbs polysaccharides	↓	Glucose ↑, Mannose ↓	Particle size ↓, Solubility ↑	Antioxidant activity	[74]
Fungal	*Saccharomyces cerevisiae*	longan vinegar polysaccharides	↓	Glucose content ↓ Rhamnose ↑	Viscosity ↓, Particle size ↓	Immunomodulatory activity	[19]
	Wine yeast	*Lycium barbarum* polysaccharide	↓			Anti-aging activity both in vivo and in vitro	[80]
	*Saccharomyces boulardii*	Chinese yam polysaccharide	↓			Antioxidant	[45]
	*Saccharomyces cerevisiae* W5	Blue honeysuckle polysaccharide	↓	Galactose ↑, Glccose ↑	Solubility ↑	Antioxidant and hypoglycemic in vitro	[69]
	*Saccharomyces cerevisiae* GIW-1	Panax ginseng polysaccharide	↓	Arabinose ↑, Galactose ↓		Antioxidant in vitro	[81]
	*Monascus purpureus*	Ginseng polysaccharide	↓			Lower blood lipid	[82]

*Schisandra sphenanthera* fermented polysaccharides (F-SSP) could significantly inhibit harmful bacteria such as *Proteobacteria* and *Escherichia-Shigella*, and increase intestinal populations of beneficial bacteria such as *Enterococcus* and *Bacteroides*, demonstrating potential prebiotic effects [82]. Soybean meal fermentation polysaccharides significantly enriched intestinal populations of *Firmicutes*, *Bifidobacteria*, and *Lactobacilli*, while decreasing the intestinal populations of *Proteobacteria* [77]. In summary, dietary polysaccharides, which largely escape host digestion, are mainly degraded in the colon by microbial CAZymes. After pre-fermentation treatment with selected strains, the *Mw*, glycosidic linkages rearrangement and substituents of polysaccharides undergo changes, which alters the recognition patterns of CAZyme, conferring a competitive advantage to bacteria equipped with matching utilization systems and driving community remodeling via cross-feeding. As a result, fermented polysaccharides generally exhibit a stronger capacity than their native counterparts to modulate gut microbiota composition and SCFAs-centered metabolic functions.

## 5. The Role of Fermented Food–Polysaccharides in Improving Various Diseases by Regulating the Gut Microbiota

Polysaccharides are often resistant to efficient degradation by host enzymes in the upper digestive tract. Instead, polysaccharides undergo progressive hydrolysis into monosaccharides primarily in the colon, relying on CAZymes (such as glycosidases and polysaccharide hydrolases) secreted by the gut microbiota. These monosaccharides are then further fermented into metabolites like SCFAs, lactic acid, and CO_2_ [83,84]. The fermentation process alters the structure and physicochemical properties of polysaccharides, thereby changing the patterns by which microorganisms recognize and utilize substrates. Simultaneously, it grants primary degraders with matching CAZyme profiles and utilization systems a competitive advantage during early utilization phases. The intermediate metabolites released by these primary degraders are then cross-utilized by secondary consumers, driving continuous reshaping of gut microbiota taxonomic composition and abundance patterns [77,85]. This process reshapes the gut microbiome and its metabolite profile (particularly SCFAs and bile acids) laying a crucial foundation for subsequent improvements in various disease phenotypes, including metabolic disorders, immune dysregulation, and intestinal injury.

### 5.1. Metabolism-Related Diseases

**Figure 3:**
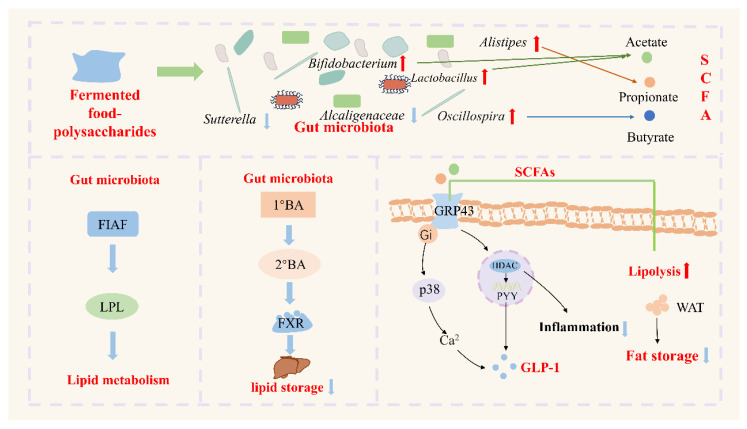
The mechanism by which fermented food–polysaccharides intervene in metabolic-related diseases by regulating the gut microbiota. Fermented food–polysaccharides reshape the gut microbiota by increasing beneficial genera (e.g., *Bifidobacterium*, *Lactobacillus*, *Alistipes*) and reducing potentially harmful taxa (e.g., *Sutterella*, *Oscillospira*; red upward vs. blue downward arrows), thereby enhancing the production of short-chain fatty acids (SCFAs: acetate, propionate, butyrate; colored lines). SCFAs activate GPR43 on intestinal and adipose cells, signaling via Gi/p38, Ca^2+^ and inhibition of HDAC to stimulate GLP-1 secretion, reduce inflammation, promote lipolysis and decrease fat storage in white adipose tissue (WAT). In parallel, the microbiota up-regulate FIAF, which inhibits lipoprotein lipase (LPL), and convert 1°BA to 2°BA that activate FXR to limit hepatic lipid accumulation.

Metabolic diseases mainly include obesity, hypertension, diabetes, and other diseases characterized by metabolic disorders. In recent years, the incidence of these diseases has been rising, posing a threat to human health and causing a huge economic burden [77]. Fermented food polysaccharides primarily regulate metabolism and improve metabolic disorders through the gut microbiota and its metabolites. Regarding the microbiota, fermented polysaccharides tend to enrich beneficial bacteria associated with lipid metabolism while suppressing potential pathogens. Fermented *Momordica charantia* L. polysaccharides (FP) significantly increased the abundance of *Firmicutes*, *Bifidobacteria*, and *Lactobacilli* in the intestines of obese rats, while reducing the proportion of harmful bacteria such as *Helicobacter pylori*, which as directly related to the regulation of lipid-related metabolic products such as fatty acid esters and eicosapentaenoic acid ethyl ester [78]. At the same time, the relative abundance of the *Bacteroidetes* and *Firmicutes* was increased. At the genus level, the relative abundance of *Enterococcus* and *Escherichia-Shigella* was decreased, while that of *Parabacteroides* and *Megamonas* was increased. Since *Bacteroidetes* possess a rich enzyme system capable of degrading polysaccharides, and the *Parabacteroides* can hydrolyze polysaccharides to promote the production of SCFAs, an increased ratio of *Bacteroidetes*/*Firmicutes* may help prevent obesity and insulin resistance [86,87,88]. Additionally, after FBP administration, the gut microbiota exhibited effects on carbohydrate metabolism (11.95%) and lipid metabolism (4.63%), indicating its potential for preventing obesity. SCFAs activates GPR43/GPR41 in L cells and pancreatic β cells, increases GLP-1, promotes insulin secretion and inhibits glucagon, thereby suppressing appetite, delaying gastric emptying, improving glucose tolerance and energy expenditure. More importantly, SCFAs and the gut microbiota can jointly regulate fat insulin signaling, thereby regulating insulin secretion and energy expenditure [89,90]. Among SCFAs, butyrate has been shown to regulate the number and function of colonic regulatory T cell populations by promoting the production of GLP-1. Butyrate can also enhance insulin sensitivity and reduce inflammatory responses in fat cells [91]. Even more, butyrate has been proven to promote fat breakdown and fatty acid oxidation, increasing the consumption of brown fat [92]. The gut microbiota also regulates insulin secretion, inhibits fat production, and promotes thermogenesis and fatty acid oxidation by metabolizing primary bile acids (1°BA) into secondary bile acids (2°BA) [93]. For example, fermented *Nostoc commune Vauch*. polysaccharides (NCVP-F) significantly upregulated the relative abundance of *Lachnospiraceae*_*NK4A136*_group and *uncultured_bacterium_f_Lachnospiraceae*, promoted bile acid metabolism into secondary bile acids, and demonstrated potential for improving metabolic diseases. Fermented food–polysaccharides can also promote the expression of fatty acid-induced adipocyte factor (FIAF) through the gut microbiota, reduce the activity of lipoprotein lipase (LPL), and enhance lipid metabolism [94]. In summary, fermentable polysaccharides exert a multi-level mechanism by reshaping the microbiota structure, promoting SCFAs and bile acid production, activating GPR41/43 and farnesoid X receptor/Takeda G protein-coupled receptor 5 (FXR/TGR5) signaling pathways, and regulating energy and lipid metabolism. This approach systematically improves insulin resistance, lipid accumulation, and chronic inflammation, thereby effectively alleviating metabolic diseases (Figure 3).

### 5.2. Immune-Related Diseases

Fermented food–polysaccharides combat immune-related diseases through tripartite modulation: restructuring gut microbiota and metabolites, regulating immunoregulatory pathways, and fine-tuning systemic immune responses. For example, fermented *Coix* seed polysaccharides (FCP) decreased the relative abundance of *Anaeroplasma* and *Peptococcaceae_unclassified* in the gut of mice exposed to high relative humidity (RH), while promoting the production of SCFAs. SCFAs not only serve as metabolic substrates for epithelial cells and erythrocytes but also function as signaling molecules, accompanied by restoration of erythrocyte Na^+^/K^+^-ATPase activity and reductions in plasma blood urea nitrogen (BUN) and ammonia (NH_3_), thereby modulating systemic nitrogen metabolism. At the immunological level, SCFAs activate GPR41/FFAR3 and GPR43/FFAR2 and inhibit HDAC activity, thereby promoting mucosal Treg expansion, suppressing Th17-mediated inflammation, modulating Th1-associated responses, and upregulating mucus and tight-junction–related genes (e.g., MUC2, ZO-1), collectively reshaping the intestinal mucosal barrier and restoring immune homeostasis [9,24]. The intervention effects of fermented *Polygonatum cyrtonema* polysaccharides (BNP) and unfermented *Polygonatum cyrtonema* polysaccharides (RNP) on cyclophosphamide (Cy)-induced immunosuppressive mouse models showed that the high-dose BNP has a more significant improvement effect on the organ indices of mice compared with RNP [94,95]. Meanwhile, BNP more effectively increased the levels of key immune indicators in serum, including tumor necrosis factor-α (TNF-α), nterleukin-2 (IL-2), and IgG, and significantly enhanced the expression of intestinal tight junction protein (zonula occludens-1, occludin). The underlying mechanism involves SCFAs acting as histone deacetylase inhibitors, thereby upregulating the transcription of tight junction proteins. In addition, BNP intervention significantly reversed the increase in the *Firmicutes*/*Bacteroidota* (F/B) ratio of the gut microbiota induced by Cy. In the Cy group, the abundances of *Lactobacillus* (47.6%), *norank_f__Muribaculaceae* (8.4%), and *Clostridium_sensu_stricto_1* (7.4%) were relatively high; after BNP intervention, the abundance of *Lactobacillus* significantly was decreased to 21.1%, and the abundance of *norank_f__Muribaculaceae* significantly was increased to 41.7%. Additionally, when fermented *Lentinus edodes* polysaccharides were used to intervene in cyinduced immunodeficient mice. it was found thatthe relative abundance of *Bacteroidetes*, *Firmicutes*, and *Fusobacteria* was increased at the phylum level; the relative abundance of *Alistipes*, *Odoribacter*, *unclassified_f__Lachnospiraceae*, and *Lachnospiraceae_NK4A136_group* was increased at the genus level [65]. Among these, *Lachnospiraceae_NK4A136_group* can reduce radiation damage, preserve the structural integrity of immune organs, and regulate immune responses by producing SCFAs. As expected, fermented *Lentinus edodes* polysaccharides promoted SCFAs production, which can inhibit histone deacetylases and suppress the activation of the nuclear factor kappa B (NF-κB) inflammatory pathway, affecting peripheral T cells, particularly Treg cells, thereby regulating the body’s immune and inflammatory responses and maintaining the microbial ecological balance of the intestinal barrier and host immune function. Additionally, SCFA reduces inflammation by regulating the NF-κB pathway, protein kinases (ERK), and other pathways [96]. SCFA can also regulate the balance between Treg and Th17 cells to improve intestinal immunity [97]. Regulating intestinal flora imbalance and intestinal barrier function damage is key to treating immune diseases. Fermented *Polygonatum odoratum* polysaccharides (PFP) increased the relative abundance of *Prevotellaceae_UCG-001*, *Muribaculaceae*, *Alistipes*, and *Candidatus_Arthromitus* in the gut. *Muribaculaceae* promoted the growth of intestinal epithelial cells, enhancing the intestinal barrier. Mechanically, SCFAs, on the one hand, activate FFAR2/FFAR3 in intestinal epithelium and immune cells. On the other hand, SCFAs enhance anti-inflammatory and antioxidant effects by inhibiting HDACs activity and increasing histone acetylation levels within the antagonistic-compensatory network involving NF-κB and nuclear factor erythroid 2-related factor 2 (Nrf2). At the same time, SCFAs promotes the amplification of mucosal Treg and upregulates barrier related genes (Muc2, Occludin), ultimately enhancing the liver’s antioxidant capacity, reducing inflammatory load, and promoting a systemic improvement in systemic immune homeostasis [98]. In summary, fermented food polysaccharides exert immunomodulatory effects by reshaping the gut microbiota and its metabolites (particularly SCFAs) through both epigenetic and receptor signaling pathways: On one hand, SCFAs repair the intestinal barrier by inhibiting HDACs and upregulating tight junction proteins (such as ZO-1, Occludin). On the other hand, SCFAs activate specific G protein-coupled receptors (GPCRs, such as GPR41, GPR43, GPR109A) to inhibit the NF-κB signaling pathway and regulate the Treg/Th17 cell balance, thereby synergistically maintaining intestinal immune homeostasis and improving systemic immune function.

### 5.3. Intestinal-Related Diseases

**Figure 4:**
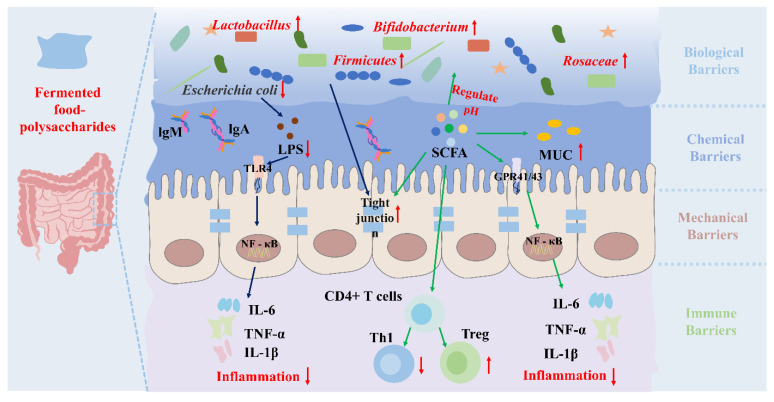
The mechanism by which fermented food–polysaccharides intervene in gut-related diseases by regulating the gut microbiota. Fermented food–polysaccharides reshape the gut microbiota by increasing beneficial bacteria (e.g., *Lactobacillus*, *Bifidobacterium*, *Firmicutes*, *Rosaceae*) and reducing pathogens such as *Escherichia coli*, thereby enhancing IgA/IgM secretion and lowering LPS–TLR4–NF-κB–mediated production of IL-6, TNF-α and IL-1β. Meanwhile, fermented food–polysaccharides elevated SCFAs and mucin (MUC) via GPR41/43 signaling tighten epithelial junctions, promote Treg differentiation, inhibit Th1-driven inflammation, and collectively strengthen the biological, chemical, mechanical and immune barriers of the gut.

Inflammatory bowel disease (IBD) is an immune-mediated disease characterized by chronic inflammation of the intestines [94,99]. Changes in diet regulate the expression of microbial genes in the intestine and reduce the incidence of intestinal inflammation [100,101]. Fermented food–polysaccharides are widely used in the treatment of intestinal diseases due to their safety, edibility, and multiple biological activities. Fermented food–polysaccharides can regulate the gut microbiota and increase the abundance of beneficial bacteria. In addition, fermented food–polysaccharides are metabolized and absorbed by the gut microbiota, producing metabolites such as SCFA that can regulate inflammation and immunity, thereby improving intestinal diseases. The specific mechanism was shown in Figure 4. Intervention with fermented soybean residue polysaccharide (FSPR) can alleviate symptoms of colitis induced by sulfate sodium salt (DSS) by markedly reducing the levels of IL-6 and TNF-α, increasing the level of IL-22, promoting the expression of E-cadherin and claudin-1, and repairing the intestinal barrier [102]. At the phylum level, FSPR increased the abundance of *Firmicutes*, while reducing the abundance of *Proteobacteria* and *Verrucomicrobia*. At the Class level, FSPR reversed the decline in the abundance of *Clostridia* and *Bacilli* caused by DSS treatment. More specifically, the abundance of *Proteobacteria* is negatively correlated with body weight. Fermented food–polysaccharides exhibit stronger regulatory effects on the gut microbiota and protective activity of the intestinal barrier compared to unfermented polysaccharides. Fermented *Hericium erinaceus* polysaccharides (FHEP) also demonstrated superior intestinal barrier protection compared to unfermented samples. by enriching *Bacteroidetes* and *Firmicutes* while depleting pathogens like *Klebsiella* and *Shigella*. This promotes SCFAs production, thereby inhibiting the NF-κB signaling pathway, reducing TNF-α, IL-1β, and IL-6 levels, and upregulating IgA, IgG, IgM, and mucin (MUC) expression to enhance barrier integrity [23]. The effects of fermented *Hericium caput-medusae* (Bull.:Fr.) Pers. polysaccharides (HFP) on the gut microbiota primarily manifested in regulating microbial composition, enhancing SCFA metabolism, and improving the intestinal microenvironment [103]. HFP significantly increaseed the abundance of beneficial bacteria such as *Ruminococcaceae* and *Fusobacteriaceae*, reduced the proportion of harmful bacteria such as *Staphylococcus* and *Enterobacteriaceae*, and enhanced the levels of SCFAs such as acetic acid, propionic acid, and butyric acid in the cecal contents. Additionally, HFP promotes Cl^−^ secretion and intestinal fluid secretion by activating chloride channels, increasing the hydration and coverage area of the mucus layer, which enhanced intestinal mucosal barrier function, reducing the risk of inflammation and infection. In summary, fermented polysaccharides remodel the gut microbiota and enhance the production of SCFAs. SCFAs in turn activate FFAR2/FFAR3, inhibit HDAC activity, and increase histone acetylation, thereby downregulating NF-κB–mediated inflammatory signaling. In parallel, fermented polysaccharides upregulate tight junction and mucus-related proteins (such as ZO-1, occludin, claudins, and MUC2) and strengthen mucosal IgA responses, jointly alleviating intestinal inflammation and restoring as well as reinforcing intestinal epithelial barrier function.

### 5.4. Other Related Diseases

In addition to affecting the aforementioned diseases, fermented food–polysaccharides can also improve conditions such as osteoporosis, food allergies, and metal-induced kidney damage by regulating the gut microbiota. The intestines are the primary route for calcium absorption in humans and mammals. Disruption of the gut microbiota can impair calcium absorption, indirectly leading to osteoporosis. Regulating the gut microbiota can modulate intestinal permeability and metabolic disorders, thereby promoting calcium absorption [104]. In a study investigating the effects of fermented *Astragalus* polysaccharides on a dexamethasone-induced osteoporosis rat model through regulation of the gut microbiota, the following results were observed: after intervention with fermented *Astragalus* polysaccharides, the relative abundance of *Lactobacillus*, *Allobaculum*, and *Blautia* was increased, while the relative abundance of *Lachnospiraceae_NK4A136_group* and *Ruminococcus* was decreased [105]. *Allobaculum* and *Blautia* promoted the production of SCFA, thereby enhancing intestinal permeability and increasing calcium absorption. Additionally, changes in the gut microbiota also promoted the production of indicaxanthin, chlorogenic acid, and 3-hydroxymelatonin, thereby enhancing calcium absorption and improving osteoporosis. Additionally, fermented food-polysaccharides influence food allergies through the gut microbiota. Treatment of food-allergic mice with fermented *Gracilaria lemaneiformis* polysaccharides increased the relative abundance of the *Firmicutes*, *Lactobacillaceae*, and *Lachnospiraceae_NK4A136_group*, while reducing the relative abundance of the *Bacteroidetes* [106]. *Gracilaria lemaneiformis* polysaccharides restored the *Firmicutes*/*Bacteroidetes* ratio, which alleviated symptoms of food allergies. These findings underscore the potential of fermented food–polysaccharides as natural bioactive compounds that offer multiple health benefits through the modulation of gut microbiota, thereby providing novel insights for their application in the development of functional foods and nutritional supplements.

## 6. Safety Assessment and Insights of Fermented Food–Polysaccharides

Although several food polysaccharides have demonstrated good tolerability in human or clinical settings (e.g., the application of *Lycium barbarum* polysaccharides in type 2 diabetes and PG2 preparations rich in *Astragalus* polysaccharides as adjuvant agents for chemotherapy), research on fermented polysaccharides began relatively late. Currently, only cytotoxicity and mouse toxicology analyses have been conducted [107,108]. On the one hand, fermentation systems themselves may generate toxins and safety-relevant impurities. For example, *Bacillus* species can produce diarrheagenic enterotoxins (nhe/hbl/cytK) and the emetic toxin cereulide, while *Aspergillus* and *Penicillium* may yield aflatoxin B_1_ (AFB_1_), ochratoxin A (OTA), or deoxynivalenol (DON) under improper piling or high-moisture storage conditions [33,109]. On the other hand, finished products require comprehensive evaluation for biogenic amines, endotoxins/cell wall components, and other trace contaminants. Additionally, experience from GRAS in the United States and QPS designations indicates that *Saccharomyces* cerevisiae and non-toxigenic, industrially bred strains of *Aspergillus niger/Aspergillus oryzae* (widely used in food enzyme preparations and ingredients), lactic acid bacteria (e.g., *Lactiplantibacillus plantarum* 299v, *L. paracasei*) and spore-formers (such as *Bacillus coagulans* GBI-30, 6086) possess long-term, well-established safety records [33,110,111]. However, GRAS/QPS judgments are inherently context-bound, applies only to specific combinations of bacterial strains, intended applications, and exposure levels. Once there is a change in the strain, application scenario, or dose, previous conclusions cannot be assumed to hold. Therefore, a systematic and long-term safety assessment of fermented polysaccharides is required.

Functionally, the effects of fermented polysaccharides observed in vitro and in animal models are likewise not uniformly beneficial. In many systems, fermentation tend to enrich polysaccharide-degrading taxa capable of recognizing specific glycan motifs (such as *Bacteroidetes* and *Firmicutes*), accompanied by increased levels of acetate, propionate, and butyrate, as well as reduced numbers of potential pathogens. However, the magnitude and direction of these shifts are jointly shaped by the interplay among substrate, microbial strain, and process parameters. Occasionally, the biological activity of polysaccharides remains unchanged or even diminishes after fermentation. For instance, in an in vitro fecal fermentation system using *Laminaria* polysaccharides, the fermented fraction (FSP) did not significantly alter gut microbial α-diversity (species richness and evenness) [112]. Following fermentation by *Lactiplantibacillus plantarum*, the hydroxyl radical scavenging capacity of yam and Lanzhou lily polysaccharides declined [74,113], and fermented okra polysaccharides showed reduced DPPH, ABTS, and •OH scavenging activity as well as weakened capacity to induce NO and IL-6 in RAW264.7 macrophages, without evidence of cytotoxicity [114]. Similarly, fermented lotus root polysaccharides (LRPs) exhibited attenuated stimulation of macrophage NO production and IL-6 secretion [115]. Potential explanations include enzymatic degradation during fermentation that disrupts bioactive conformations or critical recognition motifs, microbial consumption of synergistic antioxidant/immune-modulating cofactors (e.g., phenolic compounds), and the introduction of endotoxin or other confounders that distort in vitro immune readouts. Given that bioactivity of polysaccharide is highly dependent on structural parameters (such as molecular weight, monosaccharide composition, chain conformation, protein conjugation, and purity), future work must integrate detailed structural characterization with in vitro and in vivo functional assays to resolve structure–bioactivity relationships before and after fermentation.

From an evidence hierarchy perspective, although fermented polysaccharides repeatedly show promise in modulating metabolic disorders, immune imbalance, and intestinal inflammation in vitro and in animal models, the overall evidence remains at a mechanistically plausible but translationally unproven stage. In vitro systems cannot fully recapitulate digestion and absorption, mucosal barrier dynamics, systemic immune regulation, and endocrine signaling networks. Animal experiments, in turn, are constrained by species differences and administration routes, often using doses far exceeding realistic dietary exposure and routes (such as gavage or injection) that diverge from habitual intake, thereby risking overestimation of real-world effects. For example, fermented *Morinda citrifolia* L. (Noni) polysaccharides were administered to mice via oral gavage at a dose of 200 mg/kg·d in mice [116]. In a randomized, double-blind, placebo-controlled trial (n = 67), 300 mg/day *Lycium barbarum* polysaccharides in type 2 diabetes produced only modest reductions in postprandial glucose AUC and transient early increases in HDL [107]. A randomized, double-blind, placebo-controlled crossover study (n = 30; two 6-week intervention periods separated by a 3-week washout) showed that 15 g/day larch arabinogalactan in healthy adults modulated microbiota composition and branched-chain SCFAs, but did not significantly alter total SCFAs levels or clinically relevant endpoints [117]. PG2, an Astragalus polysaccharide injection, failed on the primary endpoint for chemotherapy-related fatigue in breast cancer patients, with only exploratory signals in premenopausal subgroups [108]. These trials commonly suffer from small sample sizes, short follow-up, inadequate preregistration of primary endpoints and correction for multiple comparisons, suboptimal implementation of intention-to-treat analyses, and insufficient embedding of mechanistic biomarkers (e.g., SCFAs and bile acids, GLP-1, and epithelial/immune transcriptional or epigenetic readouts). In parallel, incomplete reporting of product provenance and batch consistency, *Mw* and side-chain profiles, endotoxin and impurity limits further undermines reproducibility and external validity. Therefore, future human intervention and clinical trials involving fermented food–polysaccharides should be conducted with adequate sample size estimation and appropriately designed controls, while also balancing achievable human dosages and dietary feasibility. Concurrently, systematic research on the physicochemical properties and safety of fermented food-polysaccharides should be strengthened, along with comprehensive testing of relevant physiological indicators, to enhance the reliability of research findings.

Beyond safety and efficacy, it is also important to underscore that the metabolic benefits of fermented polysaccharides via microbiota reshaping do not follow a linear dose–response relationship. Instead, they exhibit significant variability influenced by dose–response characteristics and inter-individual differences. In an obese rat model, fermented Tartary buckwheat polysaccharides (FP) showed a nonlinear dose–response pattern across 50/100/200 mg·kg^−1^ (FPL/FPM/FPH). Low-dose FPL most effectively restored the *Firmicutes/Bacteroidetes* ratio and α-diversity, whereas medium and high doses (FPM/FPH) were more potent in correcting long-chain fatty acid and sphingolipid metabolism, in parallel with the enrichment of beneficial genera such as *Bifidobacterium* and *Alistipes* [72]. By contrast, in an LPS-induced colitis model, high-dose fermented *Ophiopogon japonicus* polysaccharides more markedly increased community richness and augmented beneficial taxa including *Faecalibacterium*, *Lactobacillus*, and *Bifidobacterium*, resulting in stronger anti-inflammatory effects [98]. Collectively, these findings suggest that the impact of polysaccharide interventions on gut microbiota and host metabolism emerges from the combined influence of dose, disease status, and polysaccharide structure, with distinct microbial, metabolic, and inflammatory endpoints likely corresponding to their own optimal dose. At the inter-individual level, responses to fermentable carbohydrates are more strongly determined by baseline microbial ecology than by the specific class of substrate. In trials using inulin, maltodextrin, or GOS, individuals who exhibited high butyrate concentrations in response to inulin also tended to show similar increases in butyrate levels when exposed to maltodextrin, indicating that their metabolic capacity is determined by shared characteristics reflected in their initial community structure [118]. Individuals with long-term high fiber intake and elevated baseline butyrate appear to be closer to a metabolic ceiling, leaving limited room for further improvement, whereas low-fiber/low-butyrate subjects are more likely to derive substantial benefit from fermented polysaccharides. Although the existing data primarily come from classic plant and animal polysaccharides, they have direct implications for fermented polysaccharides. Future studies should focus on more extensive safety assessments and clinical trials to determine the appropriate dosage and safety parameters for the application of fermented polysaccharides in food and related products.

## 7. Conclusions and Prospects

In summary, this paper systematically reviews the sources and fermentation process optimization strategies for fermented polysaccharides, which proposes an optimization strategy involving screening, modeling, and verification (PB→RSM, with ANN-GA employed when necessary) to determine optimal conditions. On this foundation, the fermentation strategy is implemented in an integrated optimization of “chassis–CAZymes spectrum–condition coupling”. Using on food-grade/GRAS microorganisms as the chassis, the CAZymes spectrum and metabolic flux are customized around the preset structure-functional endpoints to achieve targeted modification and controllable yield through conditional coupling. It elucidates the metabolic mechanisms of bacterial and fungal enzyme systems in terms of polysaccharides and summarizes the regulatory patterns of fermentation processes on structure and physicochemical properties of polysaccharide, including changes in *Mw*, glycosidic bond types, and branch configurations. It further analyzes the mechanisms by which fermented polysaccharides improve solubility, rheological properties, and particle structure, along with the intrinsic structure-property relationships. This paper also focuses on discussing the potential mechanisms by which fermented food polysaccharides mediate immunity, metabolism, and intestinal disease intervention through regulating gut microbiota composition and metabolites (such as SCFAs and bile acids), revealing the bidirectional regulatory relationship between fermented polysaccharides and the intestinal microbiome. There is an integrated “process–structure–property–function” theoretical framework that organically links strain selection and fermentation parameters, structural transformations of polysaccharides, and their final physicochemical properties and biological functions, forming a clear, controllable network. This provides a critical theoretical basis for rationally designing fermentation processes to prepare polysaccharide products with specific functions.

Despite the significant progress made in elucidating the relationship between structural changes in fermented polysaccharides and biological activity in recent years, existing research remains largely dependent on in vitro models. This approach has limitations, including restricted experimental scale, substantial inter-individual variability, and environmental parameters that do not align with human conditions. Future research should prioritize establishing standardized in vivo model systems to more accurately predict human gut microbiota responses to structurally diverse fermented polysaccharides. Concurrently, multi-omics integration strategies (metagenomics, metabolomics, transcriptomics) should be employed to decipher the systemic regulatory network linking fermented polysaccharides, microbiota, and hosts, revealing key structure-function sites and signaling pathways.

Furthermore, future studies should elucidate the key enzymatic pathways and core microbial communities mediating structure-specific modifications of polysaccharides, laying the foundation for rational design and targeted regulation of fermentation processes. Methodologically, high-resolution polysaccharide characterization techniques (such as high-field NMR, LC–MS/MS, MALDI-TOF, SEC-MALS) and CRISPR-based functional strain engineering strategies should be developed to enable controlled structural design and precise functional validation of polysaccharides. Additionally, research on fermented polysaccharides still faces challenges such as complex structural elucidation, microbial community heterogeneity, and in vivo mechanism clarification. However, its potential in precision nutrition and gut health regulation is opening a new chapter in the research direction of “fermented polysaccharides–gut microbiota–host health”.

## Figures and Tables

**Table 1:** Optimization methods and optimal conditions for fermented polysaccharides.

Polysaccharides	Microorganisms	Optimization Method	Optimal Condition	Evaluation Indicators	Refences
*Polygonatum kingianum* polysaccharides	*Lactobacillus plantarum* (M2)	RSM	6.6% golden pine addition, 4.7% Inoculation rate, Temperature: 36 °C	Viable bacteria count: 8.9 × 10^8^ CFU/mL	[42]
*Auricularia auricula* polysaccharides	*Trichoderma viride*	RSM	Moisture content: 61.7%, inoculation amount: 12.4%, temperature: 31.0 °C, time: 5.5 days	Degradation rate: 26.89 ± 0.14%	[43]
*Astragalus* polysaccharides (APS)	*Lactobacillus rhamnosus*	RSM	APS: 10.28%, inoculate 5.83%, time: 35.6 h, temperature: 34.6 °C	Viable bacteria count: 1.348 × 10^9^ CFU/mL	[44]
Chinese yam polysaccharides	*Saccharomyces boulardii*	Single-factor optimization method	Time: 36 h, inoculation volume: 6%, material-to-liquid ratio: 1:25 g/mL	The total reduction function (TRP) is the strongest	[45]
*Artemisia* polysaccharide	*Aspergillus niger*	RSM	Inoculation amount: 5%, temperature: 36 °C, time: 2 days, shaker speed: 180 r/min	Yield: 17.04%	[46]

## Data Availability

No new data were created or analyzed in this study. Data sharing is not applicable to this article.
